# Effectiveness of Prophylactic Doses of Tranexamic Acid in Reducing Hemorrhagic Events in Bariatric Surgery: A Systematic Review and Meta-Analysis

**DOI:** 10.1007/s11695-026-08522-7

**Published:** 2026-02-21

**Authors:** Pedro Bicudo Bregion, Josélio Rodrigues de Oliveira-Filho, Victor Kenzo Ivano, Everton Cazzo

**Affiliations:** 1 State University of Campinas, Campinas, Brazil; 2 Department of Surgery, Massachusetts General Hospital, Boston, MA USA

**Keywords:** Tranexamic acid, Hemostasis, Gastrointestinal hemorrhage, Bariatric surgery, Gastrectomy

## Abstract

Postoperative bleeding is a major complication in bariatric surgery, contributing to increased morbidity, longer hospital stays, and healthcare costs. Tranexamic acid (TXA), an antifibrinolytic agent, has shown promise in reducing surgical blood loss in various specialties. However, there is still no consensus regarding its effectiveness and safety in bariatric surgery. We performed a systematic review and meta-analysis to evaluate the impact of prophylactic TXA on hemoglobin levels, procedural duration, and hospital length of stay in bariatric surgery patients. PubMed, Embase, and Cochrane Central were searched for randomized controlled trials (RCTs) and observational studies comparing TXA and non-TXA groups in bariatric surgery up to November 2024. Data were independently extracted by two reviewers, and risk of bias was assessed using ROBINS-I and RoB-2. Outcomes were assessed using random-effects models, with heterogeneity evaluated by I2 statistics. Sensitivity analyses and publication bias assessments were also performed. Nine studies (2 RCTs and 7 observational studies) involving 1,956 patients were included. TXA significantly improved hemoglobin levels (MD 0.46 g/dL; 95% CI 0.08–0.84; *P* = 0.02), reduced procedure duration (MD -9.70 min; 95% CI -14.79 to -4.61; *P* < 0.01), and shortened hospital length of stay (MD -0.19 days; 95% CI -0.31 to -0.07; *P* < 0.01). No significant increase in thrombotic events was observed. Sensitivity analyses confirmed the robustness of these findings despite high heterogeneity. Prophylactic TXA may significantly reduce perioperative bleeding (smaller hemoglobin decline), operative time, and length of hospital stay in bariatric surgery, without a signal of increasing thrombotic risks, but this should be interpreted with caution given small sample sizes and low event rates. These results support the integration of TXA into perioperative care protocols for bariatric surgery, offering potential benefits in patient outcomes and surgical efficiency.

**Supplementary Information:**

The online version contains supplementary material available at 10.1007/s11695-026-08522-7.

## Introduction

Obesity is one of the most prevalent chronic diseases worldwide, exerting significant clinical, social, and economic impacts. Weight loss interventions often require a multidisciplinary approach, with bariatric surgery as one of the most effective long-term solutions. Among the various surgical techniques, sleeve gastrectomy has gained prominence globally due to its relative technical simplicity, the absence of anastomoses, reduced operative time, and excellent long-term outcomes [1]. However, the short postoperative course for sleeve gastrectomy is often more challenging than for gastric bypass, particularly due to late staple line bleeding and postoperative nausea [2]. Furthermore, bariatric surgery patients, many of whom require prophylactic anticoagulation to mitigate thromboembolic events, are at an increased risk for perioperative bleeding [3].

Enhanced Recovery After Surgery (ERAS) protocols are widely recognized for reducing hospital stays and improving perioperative outcomes [4]. A critical component of ERAS in bariatric surgery is minimizing intraoperative and postoperative bleeding. Over the years, various strategies have been proposed to address bleeding in bariatric procedures, including omentopexy in sleeve gastrectomy, oversewing of staple lines, the use of staplers with triple staple lines, and the placement of clips. While these techniques have shown some promise, their outcomes remain inconsistent [5].

Recently, tranexamic acid has garnered attention as an adjunct for hemorrhage control, initially popularized in obstetric settings for uterine bleeding and trauma cases [6]. Its use has since expanded to other surgical disciplines, including cystectomy and bariatric surgery, as a means of reducing bleeding complications. Despite its increasing adoption, evidence supporting the routine use of tranexamic acid in bariatric surgery remains sparse. To date, only one systematic review has been published on its application in sleeve gastrectomy, encompassing studies up to 2022 [7]. This review, although limited by a small sample size, suggested promising outcomes, including reduced postoperative bleeding without an associated increase in thrombotic events or mortality.

Following this review, several new studies have been conducted, including those assessing the use of tranexamic acid in Roux-en-Y gastric bypass and with longer follow-up durations. This updated systematic review aims to provide more comprehensive and current evidence, incorporating subgroup analyses to enhance the robustness of findings on the efficacy and safety of tranexamic acid in bariatric surgery.

## Methods

This analysis did not require ethical approval as it did not involve any human or animal subjects. The review has been registered with the National Institute for Health Research International Registry of Systematic Reviews (PROSPERO, CRD42024627867).

### Search Strategy

We performed a comprehensive literature search on PubMed, Embase, and Cochrane databases, from their inception to November 2024, to identify contemporary studies reporting prophylactic doses of tranexamic acid in reducing hemorrhagic events in bariatric surgery. We used the following search strategy in the databases: bariatric surgery AND tranexamic acid.

### Study Selection

**Fig. 1:**
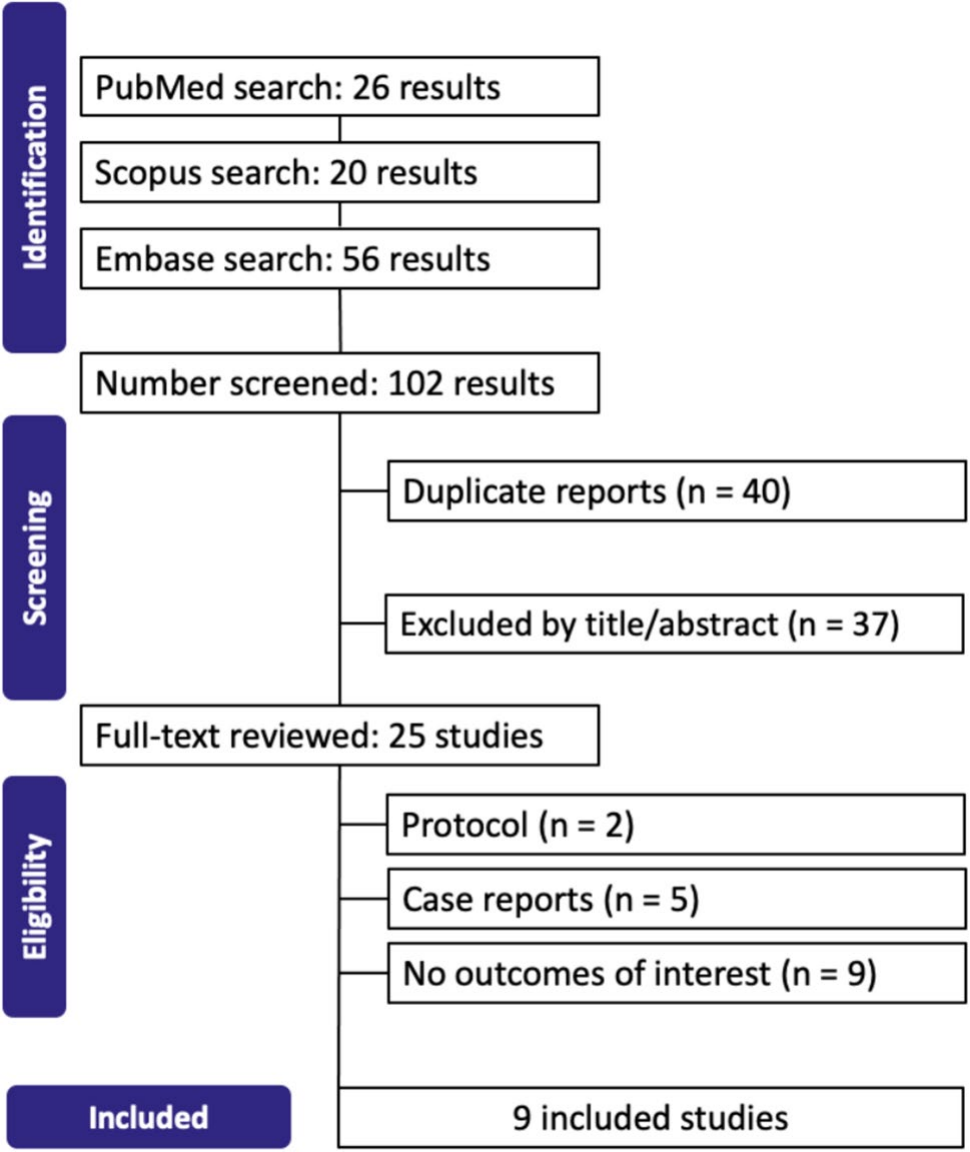
PRISMA flow diagram of study screening and selection

The study selection was conducted in accordance with the Preferred Reporting Items for Systematic Reviews and Meta-Analyses (PRISMA) guidelines, as shown in Fig. 1. Following the removal of duplicate records, two independent reviewers (JR and PB) conducted the initial screening. A third reviewer (VK) was consulted to resolve any conflicts. Titles and abstracts were assessed using predefined inclusion and exclusion criteria, and full-text versions of articles considered relevant were obtained for further evaluation.

### Eligibility Criteria

Articles eligible for this review were observational studies or randomized trials comparing procedure duration, length of stay, or change in hemoglobin levels between intervention and control groups of adult patients who underwent any bariatric surgery.

The exclusion criteria included studies without relevant outcomes for the review, conference abstracts and proceedings, case reports, and non-comparative study designs. The Supplementary Table 1 shows the quality assessment of included observational studies using the ROBINS-I, while the Supplementary Table 2 displays the risk of bias evaluation for RCTs using the Cochrane Risk of Bias 2 (RoB2) tool.

Data extraction was carried out and reviewed by two independent authors (PB and VK) using Excel spreadsheets, with a third author (JR) verifying accuracy. The extracted variables included study characteristics and patient demographics (publication year, sample size on each group, study design, time frame, age, BMI, sex, reported hypertension, reinforced staple line usage, time of administration, and TXA dosage).

### Outcomes

Primary outcomes for this review where the hemoglobin level change, defined as the difference in hemoglobin levels (g/dL) from preoperative baseline to postoperative measurements within the first 24–48 h following bariatric surgery. Intraoperative bleeding is inherently a subjective metric, with significant variability in its definition and measurement across studies. It can be quantified using several approaches, of which the most common are galanometric method, visual assessment, aspiration content, number of clips used, drain output, hemoglobin drop, reoperation due to bleeding, hematemesis or melena, or transfusion requirement.

Thrombotic events were defined as the clinical or imaging-confirmed presence of venous thrombosis at any site, including deep vein thrombosis, portal vein thrombosis, pulmonary embolism, or thrombotic acute myocardial infarction. Secondary outcomes were length of stay and operative time. Studies not reporting a given outcome were excluded from the meta-analysis for that outcome but remained eligible for other outcomes.

### Statistical Analysis

We conducted meta-analyses to compare the outcomes of prophylactic TXA and control groups in patients undergoing bariatric surgery, summarizing data from previously published studies. Mean differences (MD) with 95% CIs were utilized for every outcome. Heterogeneity among the studies was evaluated using the Cochran Q statistic and the I2 statistic. Significant heterogeneity was identified with a p-value of less than 0.05 and an I2 of 50% or higher. To account for inherent clinical heterogeneity, a random-effects model was employed. For outcomes with high heterogeneity, we performed leave-one-out analyses (Fig. 1 of the Supplementary Material). Publication bias was evaluated using funnel plots and Egger’s method for each outcome (Fig. 2 of the Supplementary Material). All statistical analyses were performed using R (version 4.4.0, R Project for Statistical Computing).

**Fig. 2:**
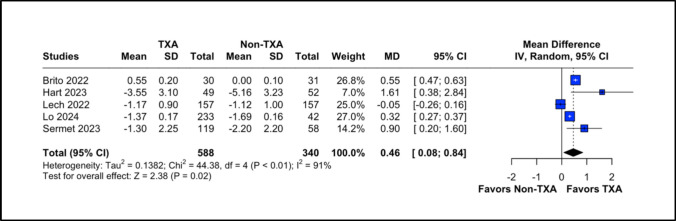
Forest plot for change in Hb levels. CI: confidence interval, MD: mean difference, Non-TXA: non- tranexamic acid, SD: standard deviation, TXA: tranexamic acid

### Heterogeneity, Sensitivity Analysis, and Publication Bias

To evaluate potential publication bias, we generated funnel plots to provide a visual assessment of publication bias. On the y-axis, we plotted the standard error (SE) of each study, with studies having higher weights (lower SE) positioned at the top. The x-axis represents the measure of association (mean difference, MD) for each study, with the vertical line in the center showing the pooled estimate from the meta-analysis. In an ideal scenario, the studies should be symmetrically distributed around the vertical line, forming a pyramid shape, with larger, higher-weight studies at the top and smaller studies at the bottom. Asymmetry in the funnel plot might indicate the presence of publication bias or small-study effects. In addition to the visual inspection of the funnel plot, we also performed Egger’s test. Egger’s test assesses the presence of publication bias in meta-analyses by examining asymmetry in the funnel plot. A *p*-value < 0.05 suggests the presence of publication bias, indicating the potential absence of smaller studies or those with negative results. Conversely, *p*-values ≥ 0.05 indicate no statistical evidence of bias, though this does not entirely exclude its possibility.

## Results

### Study Characteristics

From the systematic search, we retrieved 102 studies, out of which 9 met the inclusion criteria for the final analysis [8–16]. Figure 1 presents the PRISMA flowchart detailing the study selection process. Table 1 presents a Grading of Recommendations Assessment, Development and Evaluation (GRADE) framework summarizing the quality assessment results, while Table 2 shows the details of the included studies published between 2016 and 2024. In the final analysis, a total of 1,956 patients were included, ranging from 50 to 510.

**Table 1:** Grading of recommendations assessment, development and evaluation (GRADE) framework

Certainty assessment							№ of patients		Effect		Certainty	Importance
№ of studies	Study design	Risk of bias	Inconsistency	Indirectness	Imprecision	Other considerations	TXA	non-TXA	Relative (95% CI)	Absolute (95% CI)		
Hemoglobin levels' change (observational and RCT studies) (assessed with: mg/dL)												
5 [9, 12, 14–16]	non-randomised studies and randomized	not serious	very serious^a^	not serious	not serious	publication bias strongly suspected^b^	588	340	-	MD 0.46 mg/dL higher (0.08 higher to 0.84 higher)	⨁◯◯◯ Very low^a,b^	IMPORTANT
Hemoglobin levels' change (only RCTs) (assessed with: mg/dL)												
2 [12, 16]	randomised trials	not serious	not serious	not serious	serious^b^	publication bias strongly suspected^b^	168	110	-	MD 1.07 SD higher (0.46 higher to 1.68 higher)	⨁⨁◯◯ Low^b^	CRITICAL
Intraoperative bleeding												
5 [8–11, 16]	non-randomised studies and randomized	very serious	very serious^c^	serious	serious^c^	publication bias strongly suspected^b^	Intraoperative bleeding events were lower in the TXA groups				⨁◯◯◯ Very low^b,c^	IMPORTANT
Intraoperative Hemostasis Interventions												
6 [9–12, 16]	non-randomised studies and randomized	very serious^c^	very serious^c^	serious	serious	publication bias strongly suspected^b^	The need for clips or sutures to achieve intraoperative hemostasis was consistently lower in the TXA groups across studies				⨁◯◯◯ Very low^b,c^	IMPORTANT
Postoperative Bleeding Events												
6 [8–12, 16]	non-randomised studies and randomized	very serious^c^	very serious^c^	serious^c^	serious^c^	publication bias strongly suspected^b^	Postoperative bleeding events were markedly lower in the TXA groups				⨁◯◯◯ Very low^b,c^	IMPORTANT
Operative time (assessed with: minutes)												
7 [9–12, 14–16]	non-randomised studies and randomized	serious^d^	very serious^e^	not serious	not serious	publication bias strongly suspected^b^	873	615	-	MD 9.7 min lower (14.79 lower to 4.61 lower)	⨁◯◯◯ Very low^b,d,e^	IMPORTANT
Operative time (only RCTs) (assessed with: minutes)												
2 [12, 16]	randomised trials	not serious	not serious	not serious	serious^b^	publication bias strongly suspected^b^	168	110	-	MD 2.79 min lower (5.35 lower to 0.24 lower)	⨁⨁◯◯ Low^b^	IMPORTANT
Length of Stay (assessed with: days)												
5 [11–15]	non-randomised studies and randomized	not serious	very serious^f^	not serious	not serious	publication bias strongly suspected^b^	925	693	-	MD 0.19 days lower (0.31 lower to 0.07 lower)	⨁◯◯◯ Very low^b,f^	IMPORTANT

*CI* confidence interval, *MD* mean difference

Question: TXA compared to non-TXA for reduce hemorrhagic events in bariatric surgery

Explanations

a. I^2^ = 91%; reduced with subgroup analysis for only RCTs (I^2^ = 0%)

b. reduced number os studies

c. extensive heterogeneity: Alhomoud (2016) quantified intraoperative bleeding using the Galanometric method, which involves weighing surgical sponges and measuring aspirated fluid, and assessed postoperative bleeding through drain output combined with hemoglobin drop evaluation. Similarly, Brito (2022) [9] employed the Galanometric method intraoperatively but defined postoperative bleeding strictly as cases requiring reoperation or significant hemoglobin decline. In contrast, Chakravartty (2016), Hart (2023); Hossain (2024), and Sermet (2023) relied on visual assessments to evaluate intraoperative bleeding. These methods varied, with some focusing on the number of bleeding points along staple lines or the quantity of clips needed for hemostasis. Their definitions for postoperative bleeding also differed widely, ranging from hematemesis, melena, or excessive drain output [10]; to reoperation, transfusion, or radiological intervention. Lech (2022) employed a unique classification

d. Chakravartty with 2 domains with high risk of bias ("Bias due to deviations from intended interventions" and "Bias in selection of the reported result")

e. I^2^ = 97%; reduced with subgroup analysis for only RCTS (I^2^ = 0%)

f. I^2^ = 95%; Ommiting Deganutti 2024 led to I^2^ = 0%

**Table 2:** Demographic and clinical characteristics of the patient populations across the included studies

Study	*N* of patients		Type of study	Procedure	Time frame	Age (mean ± SD)		Female (%)		BMI (mean ± SD)		HTN (%)		Reinforce staple line	Time of administration	TXA dosage
	TXA	Non-TXA				TXA	Non-TXA	TXA	Non-TXA	TXA	Non-TXA	TXA	Non-TXA			
Alhomoud [8]	25	25	Prospective	LSG	Sep 2014 - Dec 2014	NR	NR	NR	NR	NR	NR	NR	NR	No	Induction during 5 min	10 mg/kg
Brito, 2022 [9]	30	31	Prospective	LSG	Jan 2018 - Jun 2020	37.1 ± 10.5	38.1 ± 8.6	63.3	83.9	38.9 ± 4.4	37.4 ± 2	33.3	32.3	No	Induction	1 g
Chakravartty [10]	25	25	Prospective	LSG	NR	43.1 ± 13.5	35.1 ± 9.2	80	80	53.6 ± 11.2	55.7 ± 8.4	32	16	No	Induction	1 g
Deganutti, [11]	260	250	Retrospective	LSG and LRYGB	**TXA:** May 2019 - Aug 2023 **Non-TXA:** Jan 2017 - Apr 2019	37.2 ± 5.9	40 ± 3.3	75	82	39.5 ± 2.9	41 ± 2.9	45	42	Yes	Induction	1 g
Hart [12]	49	52	RCT	LSG	2019 - 2021	36 ± 10.9	36.8 ± 12.3	77.6	80.8	42.3 ± 5.4	41.5 ± 5.3	16.3	23.1	No	Induction during 15–30 min	1.5 g
Hossain [13]	226	192	Retrospective	LSG	**TXA:** Mar 2020 - Jul 2022 **Non-TXA:** Mar 2011 - Mar 2020	39.1 ± 9.8	40.5 ± 10.3	98.7	90.6	42.1 ± 4.7	42.9 ± 5.6	NR	NR	No	Before extubation	1 g
Lech [14]	157	157	Retrospective	LSG	2016 - 2017	41.6 ± 11.5	39 ± 11.2	79	79	46 ± 9.6	44.6 ± 6.6	46.5	42.7	No	Before and after surgery	1 g
Lo [15]	233	42	Retrospective	RYGB	2018 - 2023	36.1 ± 2.6	37.2 ± 1.7	47.2	47.6	38.9 ± 0.9	37.6 ± 0.6	52.8	57.1	No	Before and after surgery	250 mg
Sermet, [16]	119	58	RCT	LSG	Feb 2022 - Aug 2022	35.6 ± 8.4	34.3 ± 9.1	78.9	77.5	42.28 ± 1.7	43.0 ± 1.6	13.4	15.5	No	Induction (TXAI) or at the end of surgery (TXAP) + both after surgery	1 g

*BMI* body mass index, *LSG* laparoscopic sleeve gastrectomy, *LRYGB* laparoscopic gastric bypass, *HTN* hypertension, *Non-TXA* non-tranexamic acid, *NR* not reported, *SD* standard deviation, *TXA* tranexamic acid

### Patient Characteristics

Table 2 provides a detailed summary of the demographic and clinical characteristics of patients in the TXA and non-TXA groups. Participants' ages ranged from 36.0 ± 10.9 years to 43.1 ± 13.5 years in the TXA group and from 36.8 ± 12.3 years to 40.5 ± 10.3 years in the non-TXA group. The proportion of female patients was comparable across groups, ranging from 47.2% to 98.7% in the TXA group and from 47.6% to 90.6% in the non-TXA group.

The mean preoperative BMI ranged from 38.89 ± 0.87 kg/m2 to 46.0 ± 9.6 kg/m2 in the TXA group and from 37.4 ± 2.0 kg/m2 to 55.7 ± 8.4 kg/m2 in the non-TXA group. The prevalence of hypertension (HTN) varied, with the highest rates observed in the TXA group at 52.78% and in the non-TXA group at 57.1%.

### TXA Timing and Dosage

The timing and dosage of TXA administration varied across studies. Most studies administered 1 g of TXA at induction, except for Hart et al. [12], which used 1.5 g over 15–30 min, and Lo et al. [15], which used 250 mg before and after surgery. Only one study administered TXA at induction over five minutes with a dose of 10 mg/kg [8]. No reinforcement of the staple line was reported in most studies, except for Deganutti et al. [11], which included staple line reinforcement. Sermet [16] had two TXA groups: one received TXA 1 g at the beginning of induction (TXAI), and the other received TXA 1 g at the end of surgery (TXAP). Both TXAI and TXAP had postoperative TXA maintenance doses.

### Prophylaxis with ENOXAPARIN

The use of enoxaparin for thromboprophylaxis varied significantly across studies, with differences in timing, dosage, and duration. Alhomoud [8] and Brito (2022) [9] did not provide specific details regarding the enoxaparin prophylaxis regimen. Chakravartty (2016) [10] administered an induction dose of 40 mg of enoxaparin and continued prophylaxis for 4 weeks postoperatively. Deganutti (2024) [11] initiated enoxaparin 12 h post-surgery and maintained it for 10 days. Hart [12] administered enoxaparin on the same day of surgery at 10:00 PM, though the dosage was not specified. Hossain [13] started enoxaparin (40 mg) 8 h post-surgery and continued it for 7 days. Lech [14] administered 40 mg of enoxaparin subcutaneously 12 h before surgery, 12 h after surgery, and once daily for 10 days after discharge. Lo [15] gave enoxaparin (30 mg) both preoperatively and postoperatively during the hospital stay, though it was unclear whether prophylaxis continued after discharge. Sermet [16] had preoperative subcutaneous injection of 40 mg enoxaparin (Clexane®) 12 h before surgery.

### Intraoperative and Postoperative Bleeding

It was not feasible to analyze intraoperative and postoperative bleeding due to the extensive heterogeneity in study definitions. For instance, Alhomoud [8] quantified intraoperative bleeding using the Galanometric method, which involves weighing surgical sponges and measuring aspirated fluid, and assessed postoperative bleeding through drain output combined with hemoglobin drop evaluation. Similarly, Brito (2022) [9] employed the Galanometric method intraoperatively but defined postoperative bleeding strictly as cases requiring reoperation or significant hemoglobin decline.

In contrast, Chakravartty [10], Hart [12], Hossain [13], and Sermet [16] relied on visual assessments to evaluate intraoperative bleeding. These methods varied, with some focusing on the number of bleeding points along staple lines or the quantity of clips needed for hemostasis. Their definitions for postoperative bleeding also differed widely, ranging from hematemesis, melena, or excessive drain output [10]; to reoperation, transfusion, or radiological intervention, for bleeding with hemoglobin levels reassessed one week later [12]; and events requiring transfusion or surgical reintervention [13].

Lech [14] employed a unique classification system for bleeding events, dividing them into three distinct categories: (I) bleeding from the staple line requiring more than eight clips for hemostasis, (II) bleeding necessitating oversewing of the staple line, and (III) postoperative bleeding severe enough to mandate reoperation. These methodological discrepancies underscore the pressing need for standardization in defining and measuring bleeding across bariatric surgery studies to facilitate meaningful comparisons and robust meta-analyses.

The number of clips used during surgery could not be statistically analyzed due to significant variability in reporting methods among studies. Some studies provided the average number of clips per patient, others reported the proportion of patients requiring more than eight clips, while certain studies only documented the number of patients who required additional measures, such as oversewing or clip reinforcement.

### Intraoperative Bleeding Outcomes

Alhomoud [8] reported intraoperative bleeding events in the tranexamic acid (TXA) group as follows: 20 out of 25 patients had bleeding volumes of less than 300 mL, 3 out of 25 had bleeding between 300–400 mL, and 2 out of 25 had bleeding exceeding 400 mL. In comparison, the non-TXA group demonstrated 5 out of 25 patients with bleeding volumes of less than 300 mL, 15 out of 25 with 300–400 mL, and 5 out of 25 exceeding 400 mL.

Brito (2022) [9] observed intraoperative bleeding volumes in the TXA group of 50 mL (range: 20–110 mL) measured via pump aspiration, with an additional 20 g (range: 10–50 g) absorbed by gauzes. The group required 11 interventions (median: 6–18) for bleeding control. The non-TXA group exhibited higher bleeding volumes of 80 mL (range: 30–300 mL) with 15 g (range: 10–50 g) gauze absorption and a lower median of 8 interventions (range: 4–24).

Chakravartty [10] reported a visual assessment of bleeding points during surgery, with 19 points in the TXA group compared to 46 in the non-TXA group, indicating a marked reduction in intraoperative bleeding with TXA use.

Deganutti [11] estimated mean intraoperative bleeding volumes of 200.1 mL ± 31.5 in the TXA group versus 270.3 mL ± 35.4 in the non-TXA group, further supporting the efficacy of TXA in reducing intraoperative blood loss.

Sermet [16] had two TXA groups (TXAI and TXAP). TXAI (induction phase) showed a median of 104 (range 1–3) bleeding points compared to TXAP (at the end of the procedure) with 132 (range 1–4) and placebo with 129 (range 1–3), demonstrating statistical significance (*p = 0.021) in favor of TXA at anesthetic induction. Intraoperative blood loss had similar results with TXAI 26.5 mL (range 10–35), TXAP 31.2 mL (range 20–35), and placebo 30.7 mL (range 15–35), with no statistically significant difference (*p* = 0.223).

### Intraoperative Hemostasis Interventions

The need for clips or sutures to achieve intraoperative hemostasis was consistently lower in the TXA groups across studies. Brito (2022) [9] reported a median of 11 interventions (range: 6–18) in the TXA group compared to 8 interventions (range: 4–24) in the non-TXA group. Chakravartty [10] documented 19 interventions in the TXA group versus 46 in the non-TXA group. Deganutti [11] observed a significantly reduced need for hemostatic interventions, with 13 cases (5%) in the TXA group compared to 35 cases (14%) in the non-TXA group. Hart [12] reported similar trends, with 34 cases (69.4%) requiring interventions in the TXA group compared to 43 cases (82.7%) in the non-TXA group.

Chakravartty [10] further detailed that only 7 patients (28%) in the TXA group required one or more stitches, while the majority (72%) did not require any intervention, representing a 33% reduction in staple line bleeding (SLB). In the control group, bleeding was more commonly observed during the second, third, and fourth staple firings (interventions: 14, 12, and 15, respectively), whereas the TXA group showed a significant reduction, particularly in the third and fourth staple firings (*p* < 0.05).

Sermet [16] demonstrated similar numbers of clips for hemostasis. TXAI required a median of 1.7 clips (range: 0–5), TXAP 1.8 clips (range: 0–4), and placebo 1.8 clips (range: 0–4), with no significant difference (*p* = 0.545).

### Postoperative Bleeding Events

Postoperative bleeding events were markedly lower in the TXA groups. Alhomoud [8] observed bleeding volumes of 50–100 mL in 20 patients, 100–150 mL in 2 patients, and 150–200 mL in 3 patients in the TXA group. Conversely, the non-TXA group had 2 patients with bleeding volumes of 50–100 mL, 16 patients with 100–150 mL, and 7 patients with 150–200 mL.

Brito (2022) [9] reported no postoperative bleeding events in the TXA group, while the non-TXA group experienced 2 cases, including one large hematoma and one reoperation due to a 300 mL bleed. Similarly, Chakravartty [10] observed no postoperative bleeding in the TXA group but documented one case of a 500 mL drain output in the non-TXA group. Deganutti [11] reported no postoperative bleeding in the TXA group, compared to 4 cases (1.6%) in the non-TXA group. Hart (2023) showed a significant difference, with 9 postoperative bleeding cases in the TXA group versus 36 in the non-TXA group.

Sermet[16] had lower drainage levels in both TXA groups: TXAI had a mean blood loss of 114.6 mL (range: 30–225), TXAP 119.4 mL (range: 30–400), and placebo 148.5 mL (range: 50–350), showing statistical significance **(***p* = 0.013).

### Hemoglobin Levels’ Change

Changes in hemoglobin levels were assessed in five studies, demonstrating a significant improvement in the TXA group compared to the non-TXA group (MD = 0.46; 95% CI 0.08–0.84; *P* = 0.02). High heterogeneity was observed (I^2^ = 91%; *P* < 0.01) and addressed through leave-one-out sensitivity analysis (Fig. 2; Supplementary Fig. 1A) and subgroup analysis. The RCT group showed I^2^ = 0%, and still favors TXA group (MD = 1.07; 95% CI 0.46–1.68; *P* = 0.32).

### Procedure Duration

**Fig. 3:**
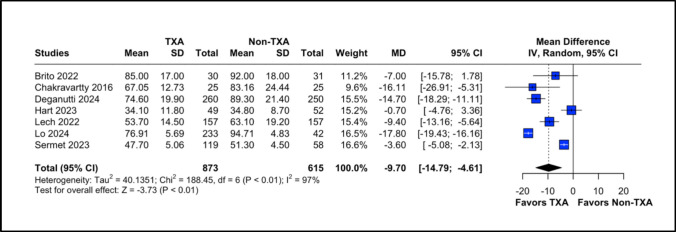
Forest plot for procedure duration. CI: confidence interval, MD: mean difference, Non-TXA: non- tranexamic acid, SD: standard deviation, TXA: tranexamic acid

Seven studies, including 1,488 patients, reported on procedure duration. The TXA group showed significantly shorter procedure duration compared to the non-TXA group (MD = −9.70; 95% CI −14.79 to −4.61; *P* < 0.01). High heterogeneity was identified (I^2^ = 97%**;** *P* < 0.01) and resolved using leave-one-out sensitivity analysis (Fig. 3; Supplementary Fig. 1B) and subgroup analysis. The RCT subgroup had lower heterogeneity (I^2^ = 42.4%) and still favors the TXA group (MD – 2.79; 95% CI −5.35 to −0.24; *P* = 0.18).

### Length of Stay

**Fig. 4:**
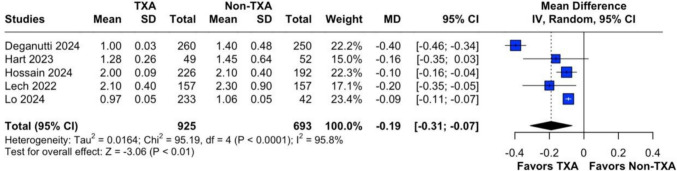
Forest plot for length of stay. CI: confidence interval, MD: mean difference, Non-TXA: non- tranexamic acid, SD: standard deviation, TXA: tranexamic acid

Length of hospital stay was evaluated in five studies comprising 1,679 patients. Patients in the TXA group experienced a shorter length of stay compared to the non-TXA group (MD = −0.19; 95% CI −0.31 to −0.07; *P* < 0.01). High heterogeneity (I^2^ = 96%; *P* < 0.01) was managed through leave-one-out sensitivity analysis (Fig. 4; Supplementary Fig. 1C).

### Thrombosis

**Fig. 5:**
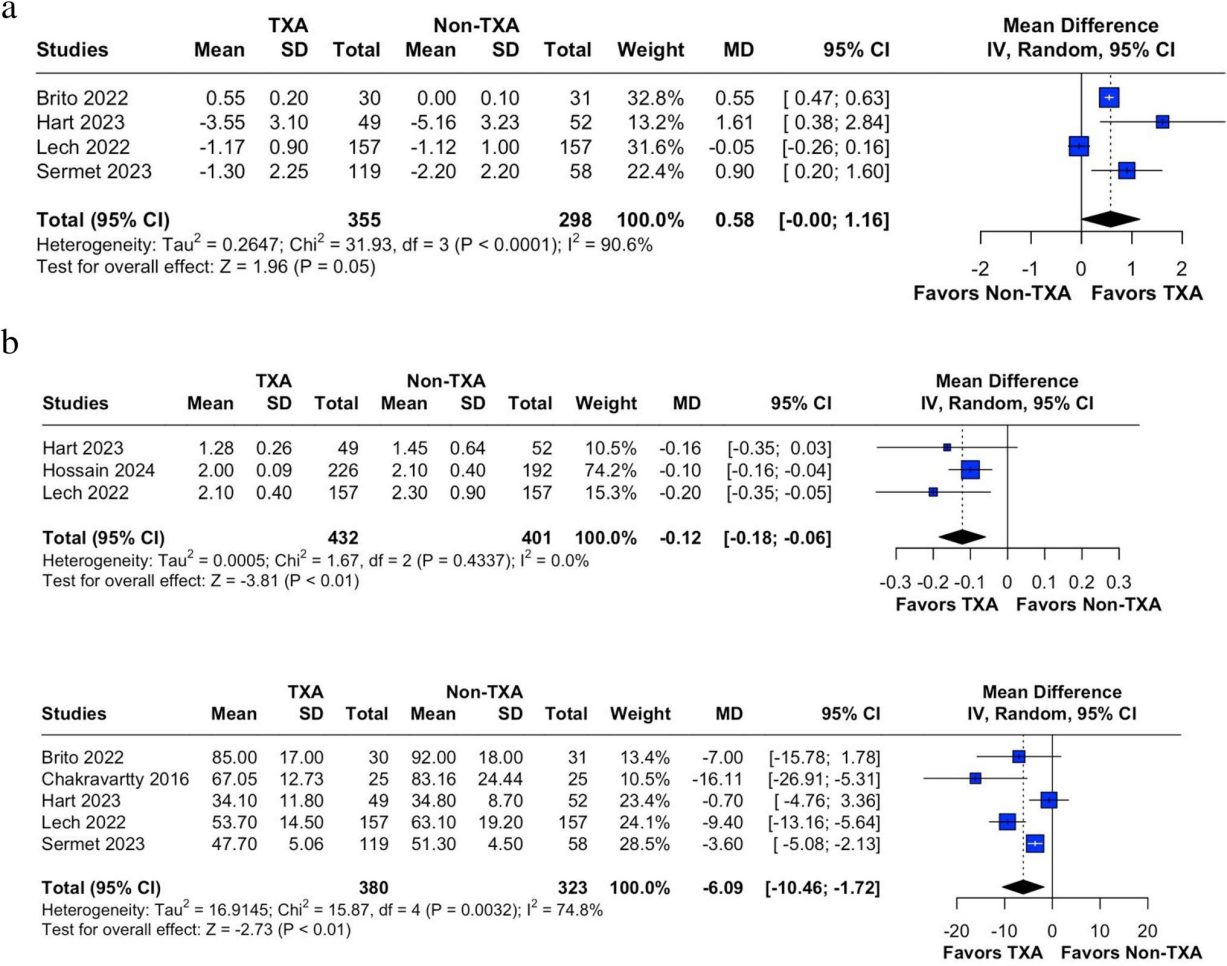
**a** Sleeve-only forest plot for change in Hb levels. CI: confidence interval, MD: mean difference, Non-TXA: non- tranexamic acid, SD: standard deviation, TXA: tranexamic acid. **b** Sleeve-only forest plot for length of stay. CI: confidence interval, MD: mean difference, Non-TXA: non- tranexamic acid, SD: standard deviation, TXA: tranexamic acid. **b** Sleeve-only forest plot for operative time. CI: confidence interval, MD: mean difference, Non-TXA: non- tranexamic acid, SD: standard deviation, TXA: tranexamic acid

Brito (2022) [9]**,** Deganutti [11], Hart [12]**,** Hossain [13]**,** and Sermet [16] specifically assessed the incidence of thrombotic events, with none of the patients experiencing thrombosis. Because several studies did not actively ascertain or report thrombotic outcomes, underreporting is possible and the true incidence of rare thrombotic events remains uncertain (Fig. 5).

### Heterogeneity, Sensitivity Analysis, and Publication Bias

For length of stay, omitting Deganutti [11] reduced heterogeneity (I2) to 0%, highlighting its significant influence due to its large sample size. However, the overall conclusion remained unchanged, favoring TXA, with a modest reduction in hospitalization duration. Sleeve-only analysis had a MD of −0.12 (95% CI −0.18 to −0.06; *P* < 0.01).

In the analysis of hemoglobin levels and procedure duration, high heterogeneity persisted despite leave-one-out sensitivity analyses. Omitting Brito (2022) [9] in the hemoglobin analysis reduced heterogeneity, though it remained high, and altered the final results to show no significant difference, with a trend favoring the TXA group. Similarly, omitting Lo [15] produced comparable findings, with no significant difference but a tendency for TXA to demonstrate benefit. We performed subgroup analysis for both hemoglobin levels and procedure duration for only RCT studies, and it reduced heterogeneity to 0%. We also performed a sleeve-only analysis (MD = −0.58; 95% CI 0.00 to 1.16; *P* < 0.01) (Supplementary material).

For procedure duration, the lowest heterogeneity was observed when omitting Lo [15], though heterogeneity remained elevated. After isolating sleeve-only studies (MD = −6.09; 95% CI −10.46 to −1.72; *P* < 0.01), the benefit in favor of TXA persisted.

Funnel plots revealed potential small asymmetry in hemoglobin levels and length of stay, suggesting possible publication bias, although Egger's test results were > 0.05 for both outcomes. In contrast, procedure duration appeared the most symmetrical, indicating less likelihood of bias.

## Discussion

Our meta-analysis highlights a compelling safety and efficacy profile for the prophylactic use of tranexamic acid (TXA) in bariatric surgery, aimed at preventing hemorrhagic events. The findings demonstrate a clear advantage for the TXA group across multiple perioperative parameters. Patients in the TXA group showed more favorable variations in hemoglobin levels, reflecting reduced blood loss, along with shorter operative times and decreased hospital lengths of stay. These outcomes underscore the potential of TXA to enhance perioperative efficiency and safety, solidifying its role as a valuable adjunct in bariatric surgery protocols. Importantly, no thrombotic events were reported in any of the included studies, even in subgroups with extended follow-up periods and in randomized controlled trials (RCTs). A single case of sleeve-related fistula was reported in the TXA group; however, no plausible mechanistic link to TXA use could be established. These results reinforce the growing utility of TXA as a practical addition to perioperative protocols, especially for high-risk patients requiring enhanced control of bleeding events.

Hemostasis, the natural mechanism to control bleeding, is a multistep process involving vascular constriction, platelet plug formation, and activation of the coagulation cascade, ultimately culminating in fibrin plug formation. TXA, a synthetic derivative of lysine, enhances hemostasis by competitively inhibiting plasminogen activation, stabilizing the fibrin clot, and preventing premature fibrinolysis [17]. This mechanism of action has been widely utilized across various surgical settings, including obstetrics, plastic surgery, trauma, and orthopedics, with significant benefits reported [18–21]. For instance, in a large meta-analysis of 54,404 patients, TXA was shown to effectively reduce postpartum hemorrhage rates, although it did result in a slight increase in thrombotic events [22]. Conversely, in trauma and orthopedic surgeries, TXA demonstrated a reduction in mortality without adversely affecting thrombotic outcomes [23]. The absence of thrombotic events in our meta-analysis suggests a distinct profile in bariatric surgery, potentially attributed to unique hemostatic dynamics in this population. However, the apparent safety of TXA with respect to thrombosis is limited by small sample sizes, low event rates, and incomplete follow-up in some studies, and there is a real potential for underreporting of thrombotic complications.

While a previous systematic review on TXA in bariatric surgery provided valuable insights, it suffered from significant methodological shortcomings [7]. For example, Alhomoud et al. [8] stratified postoperative bleeding into three categories (50–100 mL, 100–150 mL, and 150–200 mL), but the prior meta-analysis selectively reported only the highest range, potentially underestimating bleeding events. Similarly, Lech et al. [14] aggregated intraoperative and postoperative bleeding events into a single category, which likely overestimated the overall hemorrhagic risk. Our study addressed these limitations by employing stricter methodological standards, ensuring uniform data interpretation and eliminating inconsistencies that could bias outcomes.

Our study revealed a shorter operative time in the TXA group, with a mean reduction of 9.7 min (95% CI: −14.79 to −4.61), attributed to the reduced need for perioperative hemostatic revision. Additionally, the length of hospital stay was significantly lower by 0.19 days (95% CI: −0.31 to −0.07), or approximately 4.5 h. Moreover, patients in the TXA group experienced a smaller decline in hemoglobin levels between pre- and postoperative measurements, with a mean difference of 0.46 mg/dL (95% CI: 0.08 to 0.84). These findings highlight TXA’s ability to optimize surgical and recovery parameters in bariatric procedures.

Postoperative bleeding in bariatric surgery, while relatively rare (1–4%), can pose significant clinical challenges. The source of bleeding often varies by procedure. In sleeve gastrectomy, the lengthy staple line along the lesser curvature is a common culprit due to proximity to vascular structures. For Roux-en-Y gastric bypass (RYGB), bleeding often originates from intraluminal sites, particularly the gastrojejunal anastomosis. Other potential sources include the gastric pouch, excluded stomach, and mesenteric tissues. Patient-specific factors such as male sex, advanced age, lower BMI, and cardiovascular comorbidities further elevate bleeding risk. Interestingly, patients with lower BMIs may face a heightened risk due to standardized stapler heights that may not align well with reduced tissue thickness [24–31].

To minimize bleeding, various surgical strategies have been employed with varying degrees of success [32]. Oversewing the staple line is a commonly adopted measure to reinforce staple integrity, although its efficacy remains debated. The use of staplers with optimized closed heights and higher staple loading pressures has shown potential in reducing bleeding risk. Additionally, intraoperative administration of TXA has emerged as a promising adjunct, enhancing coagulation without compromising safety [32].

A critical strategy to significantly mitigate postoperative bleeding involves meticulous surgical field inspection [32]. This can be optimized by temporarily inducing higher arterial pressures, simulating pressor responses akin to those triggered by pain, and maintaining lower pneumoperitoneum pressures. These measures help identify and control bleeding that might otherwise manifest postoperatively, once tamponade effects are relieved.

In cases of significant bleeding—characterized by substantial hemoglobin drops or clinically evident symptoms such as melena or hematemesis with hemodynamic repercussions—a tailored diagnostic and therapeutic approach is necessary. This is often determined by the type of bariatric procedure performed [33]. For sleeve gastrectomy, bleeding is most frequently attributed to the staple line, making laparoscopy the preferred modality to localize and address staple line hemorrhages effectively. In contrast, in Roux-en-Y gastric bypass, the bleeding is predominantly intraluminal, likely due to sutures that invaginate the staple line into the gastrojejunal anastomosis. In such scenarios, endoscopy serves as the primary diagnostic and therapeutic tool, offering precise visualization and intervention at the anastomotic site.

The clinical and economic burden of postoperative bleeding cannot be overstated [34]. Complications such as prolonged hospital stays, intensive care monitoring, reoperations, and psychological distress significantly impact patient outcomes and healthcare systems. Financially, major bleeding events incur median costs of $5,552, while minor bleeds cost $2,406, and other techniques to minimize bleeding have failed to prove superiority (e.g. reinforced stapling)[35]. There is no formal cost effectiveness study with TXA use in metabolic and bariatric surgery, however it is highly cost-effective in trauma and postpartum hemorrhage [36]. These findings emphasize the importance of effective prophylactic strategies to mitigate these burdens.

Beyond bariatric surgery, evidence from nonbariatric general surgery aligns with our findings. In a prespecified substudy of the POISE-3 trial including 3,260 general-surgery patients, prophylactic TXA (1 g at incision and 1 g at closure) reduced bleeding versus placebo without an increase in the cardiovascular safety outcome; benefits were also observed in hepatopancreatobiliary and colorectal subgroups [37]. Consistent with this, a randomized, double-blind, placebo-controlled trial in colorectal cancer surgery showed that a single intraoperative dose of TXA reduced total blood loss and the need for transfusion without excess adverse events [38]. These data support a broader perioperative hemostatic effect of TXA and provide external context for the direction of effect we observe in metabolic and bariatric surgery, while underscoring the need for specific randomized trials with standardized outcome capture.

Most cohorts comprised sleeve gastrectomy, a setting with a plausible higher baseline bleeding risk due to thicker antral tissue and multiple staple firings. Within studies, anticoagulation protocols were consistent across TXA and control arms, making thromboprophylaxis unlikely to confound within-study contrasts. Nevertheless, between-study variation in prophylaxis schedules and reinforcement practices may contribute to heterogeneity at the meta-analytic level. A notable limitation of our analysis is the heterogeneity among primary studies, particularly regarding definitions and measurement methods for bleeding. Intraoperative assessments ranged from galanometric techniques to subjective visual scales, while postoperative bleeding definitions varied widely—from drain-based quantifications to clinically significant events requiring transfusions and reoperations. Thromboprophylaxis protocols with heparin also demonstrated variability across the included studies. Several studies did not specify whether prophylaxis was limited to inpatient care or extended post-discharge. Furthermore, variations in dosages administered and the duration of prophylaxis were observed, which could have contributed to differences in postoperative bleeding rates and overall outcomes. Reporting of thromboprophylaxis regimens (timing/dose/duration) was heterogeneous and sometimes incomplete, precluding dose- or regimen-stratified analyses. These discrepancies highlight the need for standardization in future research to ensure comparability and reliability of results. The most objective and consistent data identified in this analysis were the variations in preoperative and postoperative hemoglobin levels, length of stay, and operative time, which allowed for an adequately conducted meta-analysis. These parameters provide robust and quantifiable measures of bleeding, serving as a reliable benchmark to evaluate the efficacy of TXA in reducing perioperative hemorrhagic events. Our study contributes a standardized approach to assessing bleeding outcomes, overcoming the methodological inconsistencies that have limited previous research in this field.

Our findings underscore TXA’s potential as a transformative addition to perioperative bariatric care protocols. By reducing bleeding complications without elevating thrombotic risks, TXA offers a safer, more efficient approach to managing postoperative hemorrhagic events. However, further high-quality RCTs with standardized methodologies are necessary to validate these findings and facilitate direct comparisons with other interventions, such as oversewing or alternative pharmacological agents. This study not only supports TXA’s current application but also opens avenues for future research aimed at optimizing perioperative outcomes in bariatric surgery.

## Conclusion

Tranexamic acid (TXA) demonstrates a robust safety and efficacy profile in bariatric surgery, effectively reducing postoperative bleeding without increasing thrombotic risk. Our meta-analysis underscores its potential as a key perioperative intervention, particularly for high-risk patients prone to hemorrhagic complications. By addressing a critical gap in bariatric care, TXA provides a safer, efficient alternative, paving the way for improved surgical outcomes and setting the foundation for future standardized protocols. Accordingly, our safety findings should be interpreted cautiously in light of small samples, low event rates, variable and sometimes incomplete follow-up, and the possibility of underreporting, underscoring the need for larger RCTs with standardized, longer follow-up.

## Supplementary Information

Below is the link to the electronic supplementary material.

Supplementary file1 (DOCX 905 KB)

## Data Availability

No datasets were generated or analysed during the current study.
