# Supplementary material for: Effectiveness of Prophylactic Doses of Tranexamic Acid in Reducing Hemorrhagic Events in Bariatric Surgery: A Systematic Review and Meta-Analysis
Source: Obes Surg. 2026 Feb 21;36(4):1876–89. doi: 10.1007/s11695-026-08522-7 (PMC13083335; doi:10.1007/s11695-026-08522-7)
Supplement: Supplementary file 1 — Supplementary file1 (DOCX 905 KB) [file 11695_2026_8522_MOESM1_ESM.docx]

**Supplementary Material**

**Supplementary Table 1.** Assessment of risk of bias using the Risk Of Bias In Non-Randomized Studies - of Interventions (ROBINS-I).

**Supplementary Table 2.** Assessment of risk of bias using the Cochrane Risk of Bias 2 (RoB2) tool.

**Supplementary Figure 1**. Leave-one-out analyses for all outcomes.

**Supplementary Figure 2.** Publication bias using funnel plot and Egger’s method for all outcomes.

**Supplementary Table 1.** Assessment of risk of bias using the Risk Of Bias In Non-Randomized Studies - of Interventions (ROBINS-I).


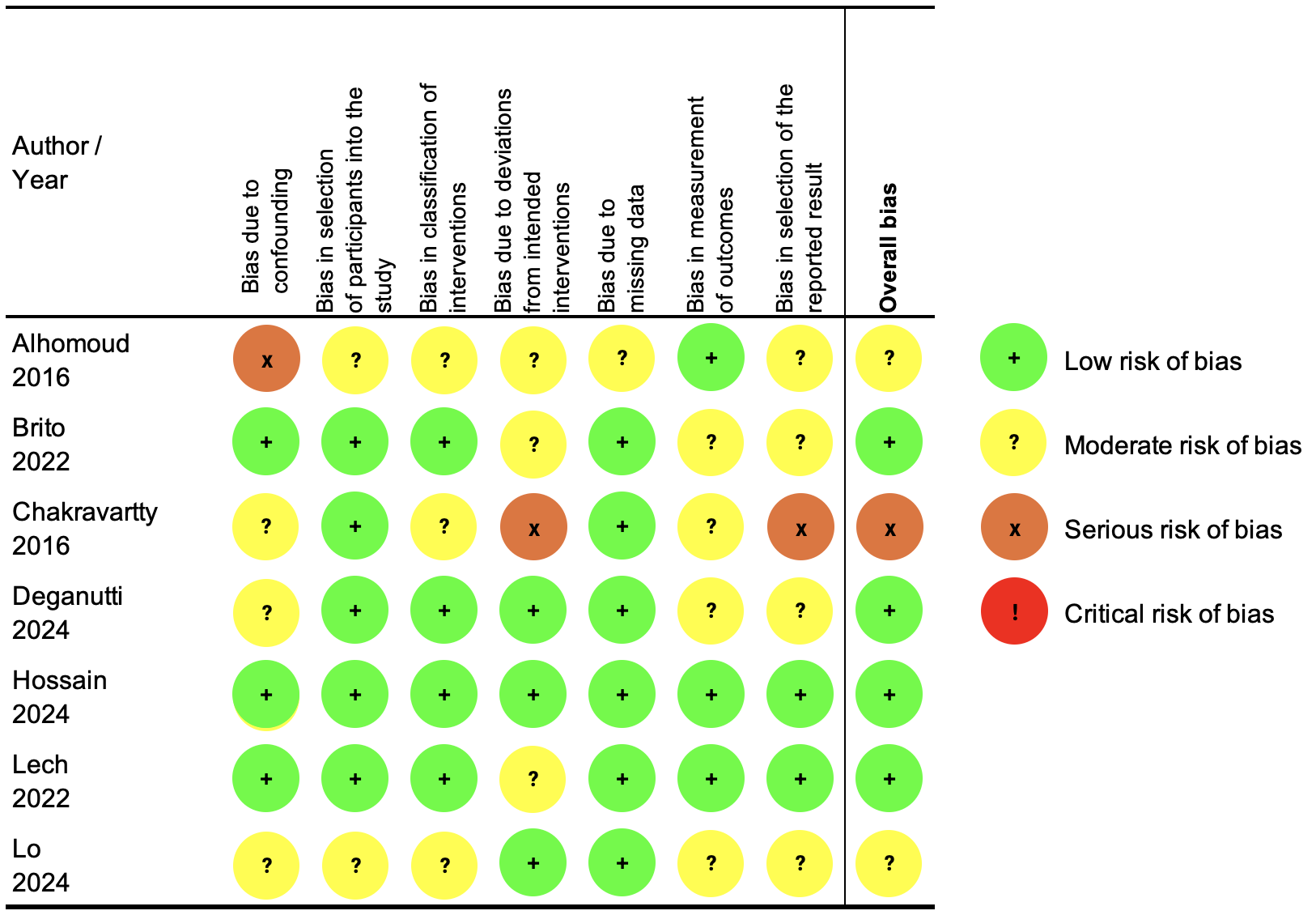


**Supplementary Table 2.** Assessment of risk of bias using the Cochrane Risk of Bias 2 (RoB2) tool.


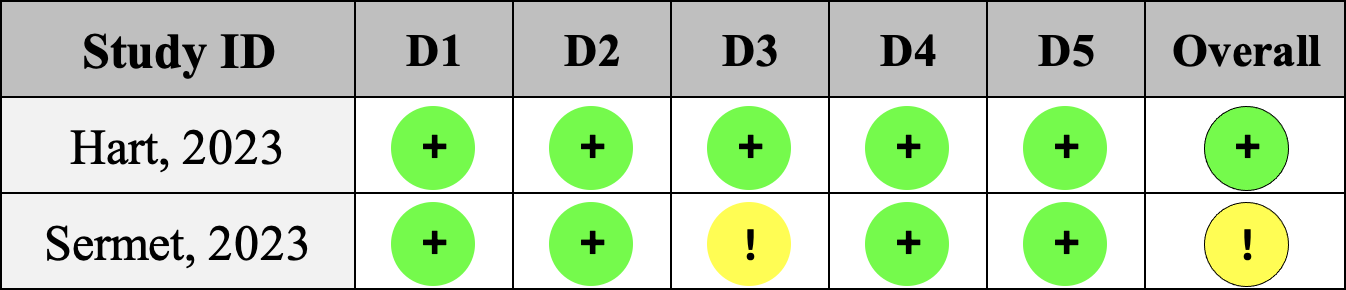


**Supplementary Figure 1**. Leave-one-out analyses for all outcomes. **Figure 1A** shows Leave-one-out analysis for change in Hb levels**. Figure 1B** shows Leave-one-out analysis for procedure duration **Figure 1C** shows Leave-one-out analysis for length of stay.

**
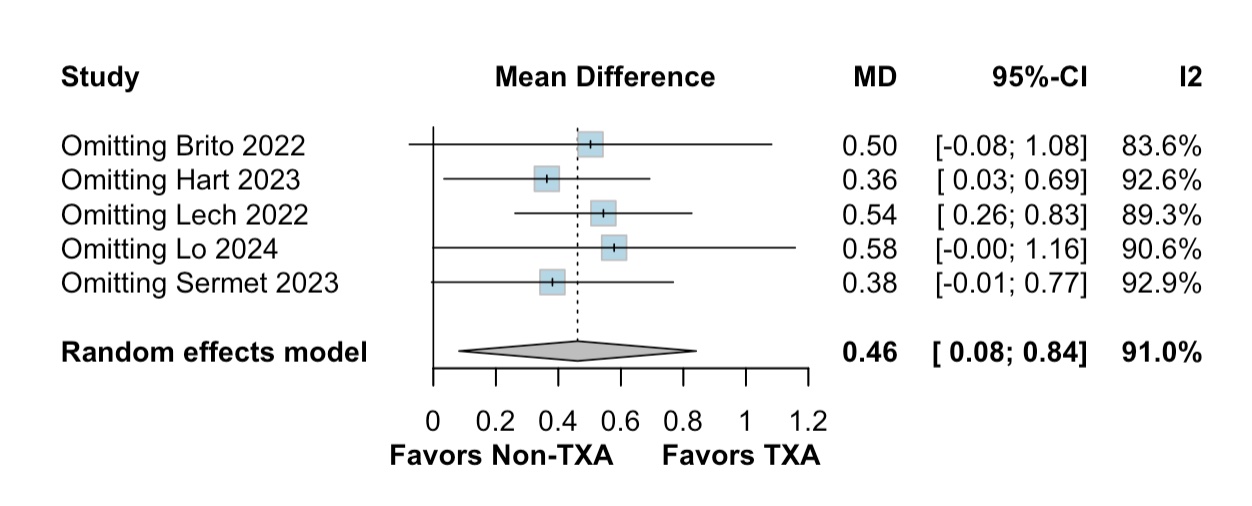
**

**Figure 1A** shows Leave-one-out analysis for change in Hb levels**.**

**
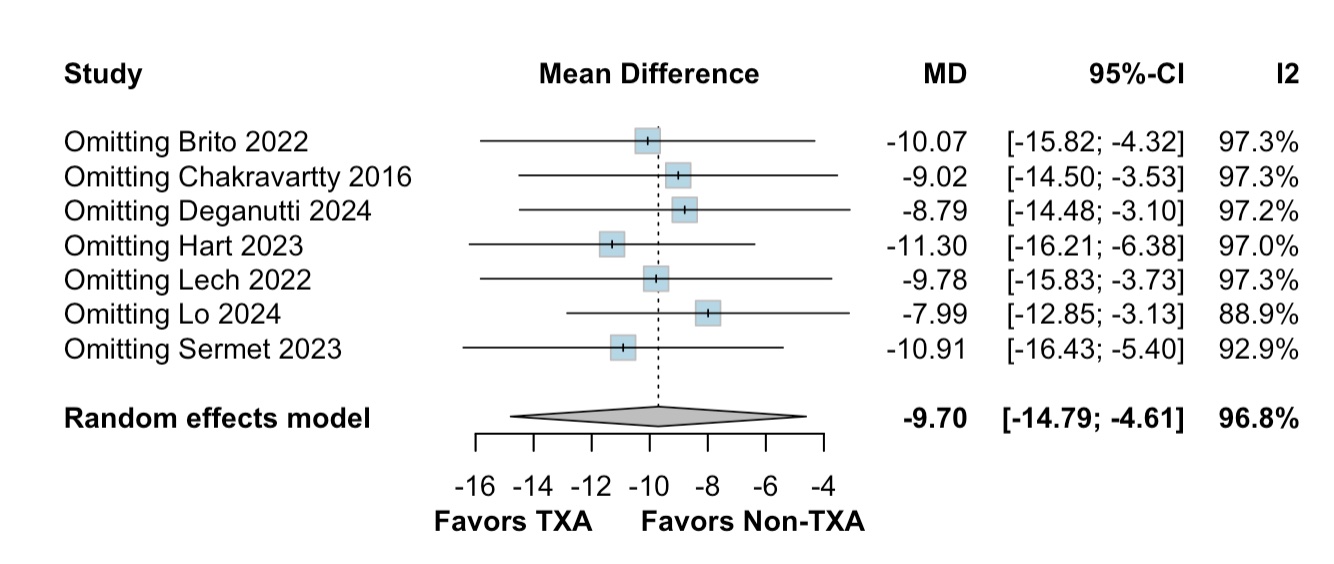
**

**Figure 1B** shows Leave-one-out analysis for procedure duration


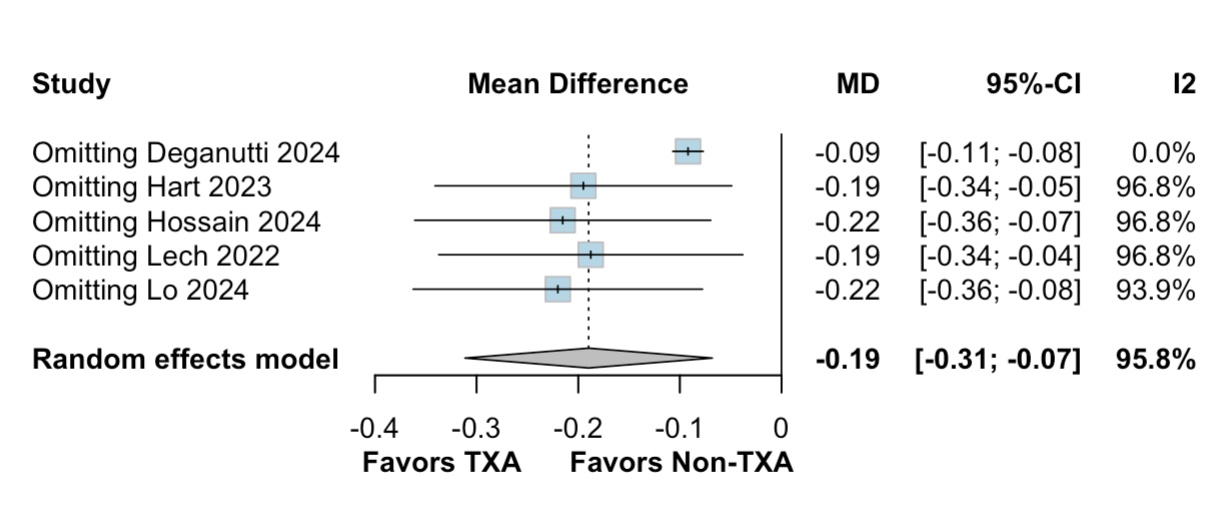


**Figure 1C** shows Leave-one-out analysis for length of stay,

**Supplementary Figure 2.** Publication bias using funnel plot and Egger’s method for all outcomes. **Figure 2A** shows funnel plot for change in Hb levels**. Figure 2B** shows funnel plot for procedure duration. **Figure 2C** shows funnel plot for length of stay.

**
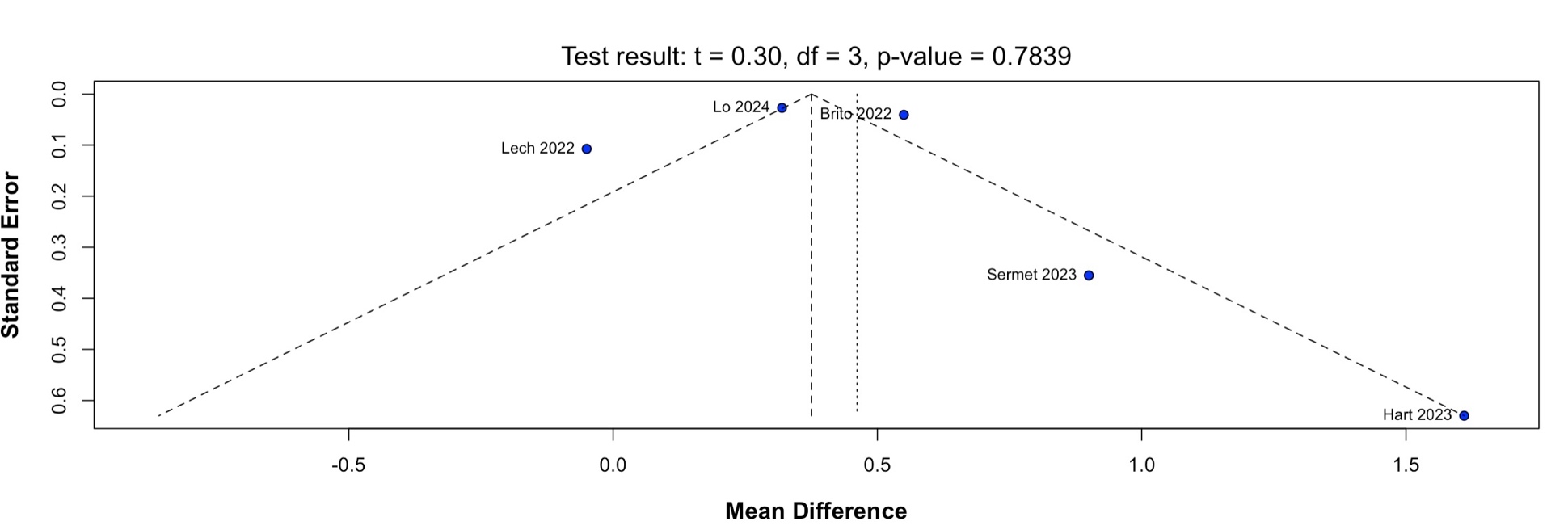
**

**Figure 2A** shows funnel plot for change in Hb levels**.**


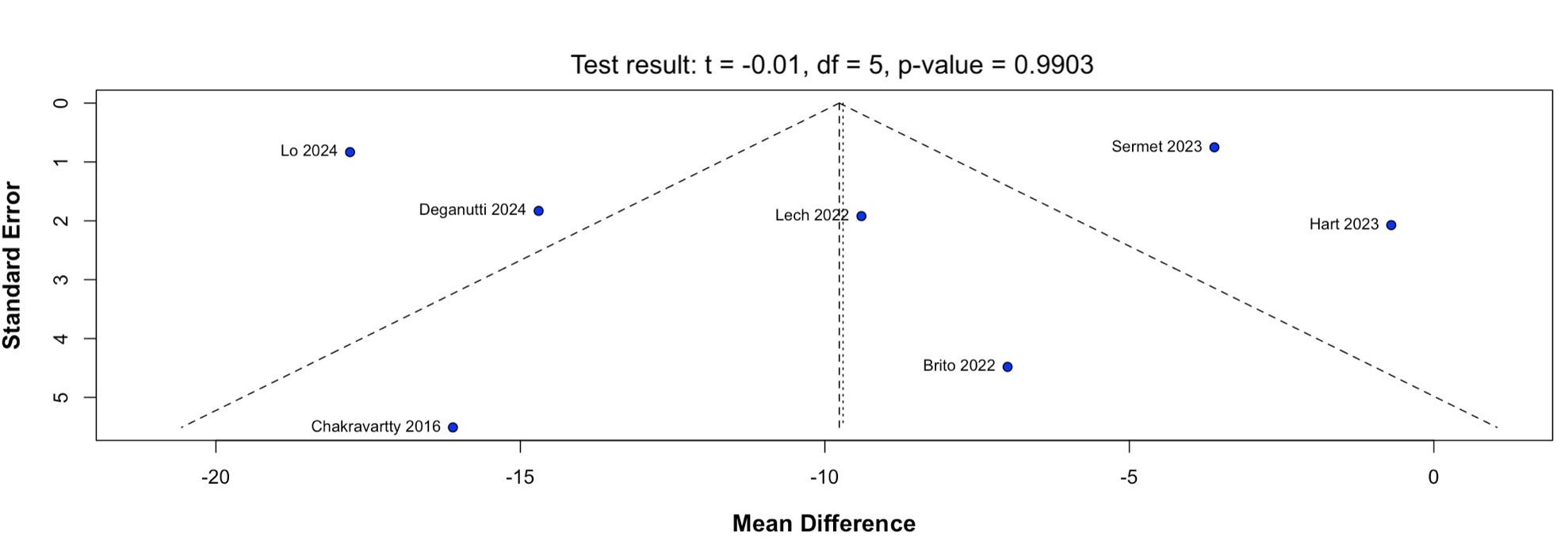


**Figure 2B** shows funnel plot for procedure duration.


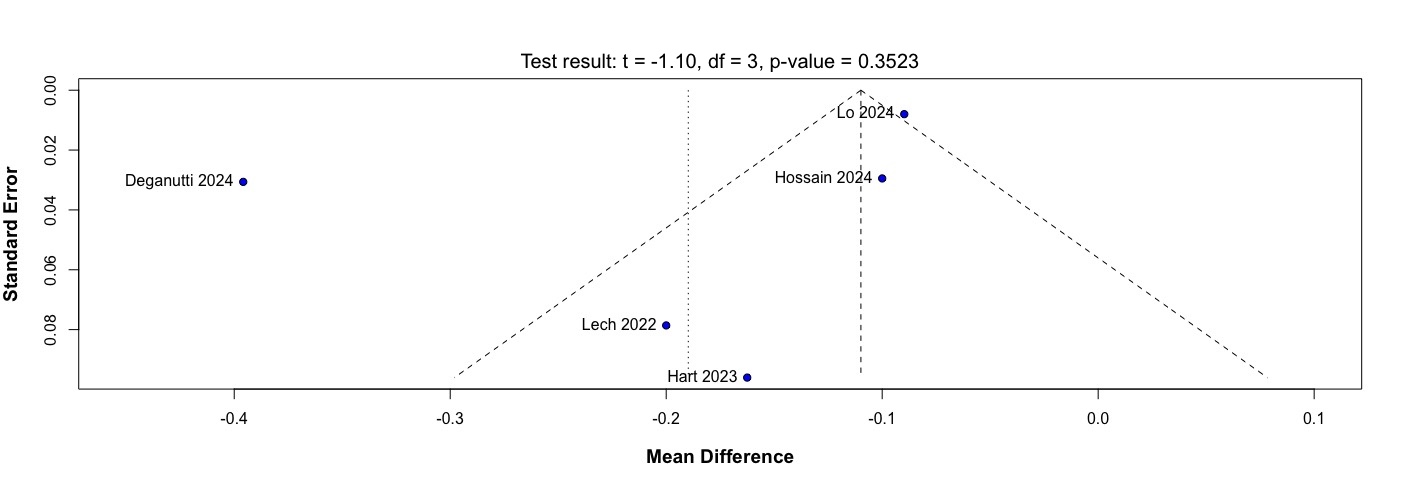


**Figure 2C** shows funnel plot for length of stay.
